# SARS-CoV-2 infection: can ferroptosis be a potential treatment target for multiple organ involvement?

**DOI:** 10.1038/s41420-020-00369-w

**Published:** 2020-11-25

**Authors:** Ming Yang, Ching Lung Lai

**Affiliations:** 1 Department of Ophthalmology, Li Ka Shing Faculty of Medicine, The University of Hong Kong, Hong Kong, China; 2 Department of Medicine, Li Ka Shing Faculty of Medicine, The University of Hong Kong, Hong Kong, China

**Keywords:** Microbiology, Cell death

## Abstract

Since the outbreak of the new coronavirus in 2019 (SARS-CoV-2), many studies have been performed to better understand the basic mechanisms and clinical features of the disease. However, uncertainties of the underlying mechanisms of multiple organ involvement remain. A substantial proportion of severe coronavirus disease 2019 (COVID-19) patients have lymphopenia, low serum iron levels, and multiple organ involvement. Several therapeutic agents have been used for different stages of the disease, but the treatment for severe disease is still suboptimal. Understanding the mechanism of programmed cell death in COVID-19 may lead to better therapeutic strategies for these patients. On the basis of observations of basic science studies and clinical researches on COVID-19, we hypothesize that ferroptosis, a novel programmed cell death, may be an important cause of multiple organ involvement in COVID-19 and it might serve as a new treatment target. In spite of the existing findings on the involvement of ferroptosis in SARS-CoV-2 infection, there is no reported study to uncover how does ferroptosis acts in SARS-CoV-2 infection yet. Uncovering the role of ferroptosis in SARS-CoV-2 infection is essential to develop new treatment strategies for COVID-19. Intracellular cell iron depletion or new generation of ferroptosis inhibitors might be potential drug candidates for COVID-19. We hope this hypothesis may launch a new wave of studies to uncover the association of ferroptosis and SARS-CoV-2 infection in vitro and in vivo.

## A snapshot of COVID-19

Ever since January 2020, clinical studies have been reporting the features of severe acute respiratory syndrome coronavirus 2 (SARS-CoV-2) globally. The general symptoms of the new coronavirus infection are fever, fatigue, and dry cough. Some patients have mild symptoms or are asymptomatic. However, severe symptoms may develop, including acute respiratory distress syndrome, septic shock, marked metabolic acidosis, and coagulopathy. The majority of patients have a good prognosis, but around 20% of patients have severe disease with high risk of death.

Most patients with mild disease have slight or no residual dysfunctions^1–3^. However, for severe or critically ill patients, there may be persistent shortness of breath with limited physical activities due to pulmonary fibrosis as revealed by chest computed tomography examination^2–4^.

Angiotensin-converting enzyme 2 is widely expressed in multiple organs and serves as a critical receptor of the spikeRB receptor-binding domain on the surface of SARS-CoV-2 for invasion of the respiratory tract^5,6^. Several pathophysiological mechanisms have been proposed. These include (i) direct virus-induced cell damage, (ii) dysregulation of the renin–angiotensin–aldosterone system, and (iii) cell damage of endothelial layer and thromboinflammation, and imbalance of immune response and inflammatory cytokine storm, leading to multiple organ failure (MOF) syndrome^7^.

## COVID-19 and multiple organ involvement

A large number of studies have reported distinctive multiple organ involvement in coronavirus disease 2019 (COVID-19) patients^2,8–11^.

Even with respiratory failure, the most common cause of death, there are histologic features of the lungs that are unique. In a study of seven lungs at autopsy, Ackermann et al.^12^ found that, other than diffuse alveolar damage and perivascular lymphocyte infiltrates, which are also seen in the lungs of patients with influenza A (H1N1), there were three angiocentric features associated with COVID-19 patients as follows: severe endothelial injury with intracellular SARS-CoV-2 virus and disrupted endothelial cell membranes; widespread microangiopathy with vascular thrombosis; and new vessel growth through intussusceptive angiogenesis. Intussusceptive angiogenesis may be related to the greater degree of endothelialitis and thrombosis in COVID-19 patients.

Acute myocardial injury (12% in 41 patients) and arrhythmia (44.4% in 36 patients requiring intensive care unit (ICU) admission) have been reported^8,10^. Acute kidney injury also occurs, which may be related to the direct effects of the virus, hypoxia, or shock^10^. Liver derangement with elevations of serum alanine aminotransferase and aspartate aminotransferase is reported in 16–53% of patients^8,11^. However, serum alkaline phosphatase is normal in most patients. It is postulated that the liver derangement may be due to underlying chronic liver disease (including hepatits B, hepatitis C, and steatohepatitis), sepsis-related inflammatory changes, or drug hepatotoxicity^13^.

In a review of thrombosis risk in COVID-19 patients, venous thromboembolism and stroke occurred in 20% and 3% of patients respectively^14^. It is postulated that disseminated intravascular thrombosis in COVID-19 patients may represent a distinct entity of coagulopathy. The proportion of venous thromboembolism, including pulmonary embolism, is much higher in severe disease with patients requiring ICU admission. Arterial thrombosis causing stroke and acute coronary syndrome/myocardial infarction also occurs, mainly in patients with severe disease^15^.

As SARS-CoV-2-induced damage in multiple organs increases the risk of poor prognosis, it is necessary to understand the critical mechanisms that contribute to the damage,to decrease the occurrence of MOF.

## Ferroptosis as a new form of programmed cell death

The term “ferroptosis” was first proposed in 2012^16^. Unlike apoptosis, it is a newly discovered programmed cell death, which is mainly caused by the accumulation of lipid reactive oxygen species (ROS) in cells, resulting in fatal lipid peroxidation^17^. As ferric ion overload is the primary factor leading to the accumulation of lipid ROS, it is named iron death.

Ferroptosis can be classified into canonical and non-canonical types to date. Canonical ferroptosis is started with the failure of glutathione peroxidase (GPX4) defense, leading to excessive lipid peroxidation and cell death^18^. Inactivation of GPX4 and glutathione (GSH) depletion play a central role in the induction of canonical ferroptosis^18,19^. Iron (II) oxidizes lipids in the Fenton reaction (hydrogen peroxide, iron and lipid) is the hallmark feature of ferroptosis, thereby generating lipid ROS, causing cell membrane damage^17–19^. GPX4 eliminates lipid ROS by consuming GSH and protects cell membrane against lipid peroxidation and ferroptosis^18^. Moreover, cystine/glutamate transporter (xCT) is responsible for providing cystine to produce GSH for GPX4 to function normally. Iron is an important metal in cells. But there is no efficient mechanism for excreting iron in the human body and, as a result, iron homeostasis is vulnerable to stresses.

In 2018, Hassannia et al.^20^ introduced the concept of non-canonical ferroptosis induction. They demonstrated iron overload-induced increase in intracellular labile iron (II) pool, accompanied with the excessive activation of heme oxygenase-1, is sufficient to induce non-canonical ferroptosis^20^. In general, four key factors determine ferroptosis as follows: (i) iron overload in cells, (ii) decrease expressions of GPX4 and xCT, (iii) the activation of acyl-CoA synthetase long-chain family member-4 (ACSL4) and lysophosphatidylcholine acyltransferase-3, and (iv) the final increase of lipid peroxidation.

Lipid peroxidation has enzymatic (enzymatic oxygenation) and non-enzymatic (free-radical chain reaction) types. Enzymes such as lipoxygenases (LOXs), non-heme iron dioxygenases, determine the peroxidation that triggers ferroptotic cell death^21,22^. The non-enzymatic type is driven by carbon- and oxygen-centered radicals^23^. Ferroptosis could be induced through iron-dependent free-radical mechanisms, which is a non-enzymatic lipid peroxidation^24^. Both types are quenched by the GSH/GPX4 axis^25^ and produce lipid peroxide degradation product such as 4-hydroxynonenal (4-HNE) and malondialdehyde, serving as the detection markers of ferroptosis^26^.

The execution of ferroptosis is peroxidation of phospholipid species. Recently, it has been shown that LOXs could directly oxidize arachidonoyl (AA) and adrenoyl (AdA) phosphatidylethanolamines (PE), thereby triggering ferroptosis^21^. The transformation of AA and AdA into PE is ACSL4-dependent in this process. ACSL4 ligates coenzyme A to long-chain polyunsaturated fatty acids, which esterifies lysophospholipids in the cell membrane to undergo lipid peroxidation and ferroptosis^21,27^. Apart from these ways, the breakdown of lipid peroxides generates 4-HNE, which is also considered to execute ferroptosis^28^.

To date, in vivo studies have addressed the immunogenic effect of ferroptosis, apart from boosting innate immunity^20^. Inhibiting ferroptosis prevents certain diseases by anti-inflammatory mechanisms^29^. Ferroptosis has been shown to contribute important pathogenic roles in multiple system diseases involving the heart, the liver, the gut, the lungs, the kidneys, and the nervous systems^18^. It has been proven to be a primary mechanism in *Myobaterium tuberculosis* infection causing damage to various organs of the host^30^. Lipid peroxidation of T-cell causes ferroptosis, thereby preventing the infection of the immune system^31^. Iron is essential for pathogens to proliferate. Iron overload is an important mechanism contributing to the pathogenesis of various viruses such as hepatitis B^32^, hepatitis C^33,34^, HIV-1^35^, and human cytomegalovirus infection^36^. Reducing the iron level in the infected cells can effectively inhibit the growth of these viruses and development of the diseases induced by the viruses^37,38^. These studies suggest the direct manipulation of iron metabolism by viruses and the consequences of iron homeostasis imbalance caused by viruses. As the outcome of ferroptosis is cell death, it might explain the clinical features of multiple organ involvement and failure in COVID-19 patients.

In addition, the induction of ferroptosis in cancer cells can promote the death of cancer cells. This discovery brings new ideas for the development of anti-cancer drugs, especially for drug-resistant cancer cells, providing an alternative choice to induce cancer ferroptosis^39,40^.

Iron accumulation happens in the infected cells, while iron deprivation decreases the survival of the pathogens^38^, perhaps even the coronavirus^37,41^. This may lead to more effective treatment options for COVID-19.

## A hypothesis on the association between COVID-19 and ferroptosis

Iron is considered a critical player in COVID-19 pathogenesis^42^. A basic study used patient-derived SARS-CoV2 (SZ005) to infect African green monkey kidney (Vero) cells and found the expression of GPX4 was significantly decreased in mRNA levels, suggesting the association between SARS-CoV-2 and ferroptosis^43^. Owing to the lack of GPX4, GSH cannot be peroxidated to reduce lipid ROS generated from Fenton reaction. As a result, lipid ROS accumulation would cause lipid peroxidation and ferroptosis. Clinical studies show that patients with severe COVID-19, who had no history of underlying kidney diseases, could develop kidney malfunction or damage^44–46^. Therefore, it is possible that the ferroptosis contributes to the renal manifestations of COVID-19. Iron accumulation takes time and this is probably why COVID-19-induced damages usually appear at 10–14 days.

Iron metabolism is dysregulated in COVID-19 patients^47,48^. A clinical study^49^ enrolled 50 patients diagnosed with COVID-19 and investigated their serum iron levels. They found that 90% of the patients had low serum iron levels. The serum iron levels were negatively associated with the severity of the infection. This was further confirmed by comparing the serum iron levels prior to the treatment with post-treatment levels, with the latter being relatively higher. Another study^47^ involved 222 patients and 88.2% of them had dyshomeostatsis with functional serum iron deficiency, suggesting anemia is prevalent in COVID-19 patients. Transferrin is the carrier of iron, transporting it through the membrane and reach to the cytoplasm^18^. In this study, both serum iron concentration and transferrin were significantly lower in the anemic patients.

The absorption from the intestine and the degradation of erythrocytes produce Fe^2+^, which is oxidized into Fe^3+^ through ceruloplasmin^48^. The Fe^3+^ generated can further bind to membrane transferrin thereby entering the cells^18,48^. Iron overload is the primary cause of ferroptosis^16^. Virus replication requires iron to serve as a raw material, as with human immunodeficiency virus, cytomegalic virus, vaccinia virus, herpes simplex virus 1, and hepatitis B virus^38^. Therefore, it is likely to be that more iron will be transferred into the cell, triggering Fenton reaction and generating excessive lipid ROS, which cannot be eliminated by the reduced amount of GPX4. Consequently, ferroptosis may occur due to the dysregulation of iron homeostasis in COVID-19 patients.

Iron enters into the cell through transferrin receptor^48^, which also serves as an ideal portal for various microorganisms to enter cells^50^. A study showed that the expression of transferrin was age-related and male had significant higher levels of expression compared with females^51^. This is associated with the severity of COVID-19 with the mortality of male and elderly patients was higher than that of female and young patients^1^. Recent clinical studies showed serum ferritin levels were significantly higher in severe COVID-19 patients, with hyper-ferritinemia compared with the mild cases^52,53^. Hyper-ferritinaemia is a condition in which excess ferritin accumulates in the body. Upon SARS-CoV-2 infection, interleukin-6 in the cytokine storm increases ferritin and the production of hepcidin, which plays a key role in iron regulation. As iron is sequestered by hepcidin in the enterocytes and macrophages, intracellular ferritin will be increased, leading to a decreased iron efflux from the cells^54^. Therefore, the hyper-ferritinemia in COVID-19 patients is likely caused by cell death and tissue damage, thereby releasing the intracellular ferritin^42,55^. High serum iron might be followed by low iron depending on the stage. However, the dynamic fluctuations of hepcidin require further study.

Recent studies showed that labile iron originates from the degradation of ferritin through autophagy, which is termed as “ferritinophagy”^56,57^. Ferritinophagy requires nuclear receptor coactivator 4, which is a selective carrier receptor that transports ferritin to the autophagosome^56,58^. Ferritinophagy facilitates ferroptosis through degradation of ferritin^59^ or the bromodomain protein BRD4 inhibitor (+)-JQ in cancer cells^60^. Also, the accumulation of intracellular ferritin may indicate the activation of ferritinophagy^61^. However, further studies should be performed to evaluate whether NCOA3 knockdown/knockout could enhance this accumulation to confirm the involvement of ferritinophagy in SARS-CoV-2 infection.

**Fig. 1:**
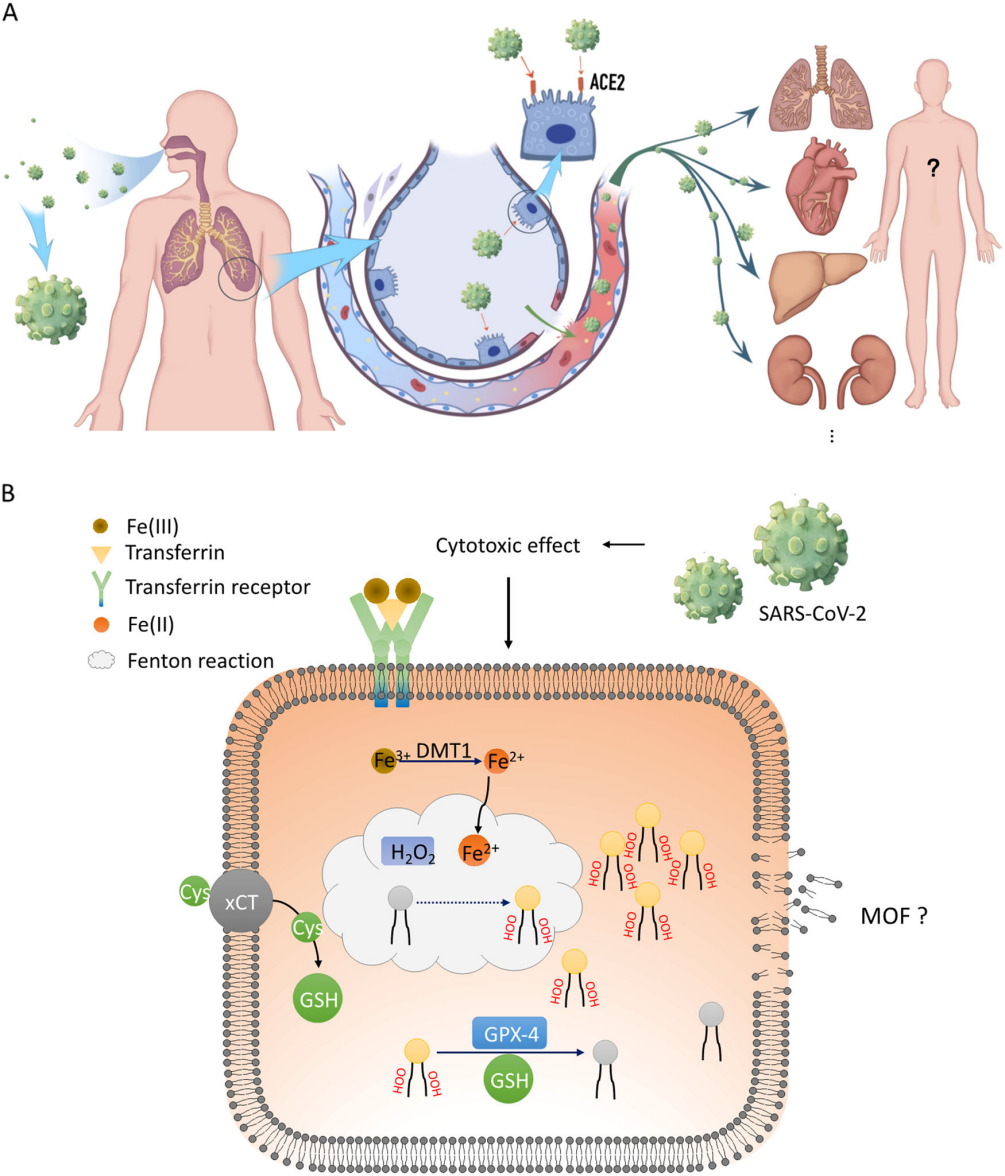
SARS-CoV-2, Multiple Organ Failure and the Possible Triggering of Ferroptosis. **A** Schematic representation of SARS-CoV-2 causing multiple organ failure (MOF). SARS-CoV-2 recognizes the AEC2 receptor in the alveoli, especially the type II alveolar cells (AT2). This infection triggers the immune response and inflammation, causing damage to the blood-air barrier. In this case, SARS-CoV-2 therefore passes through the barrier, reaching to the capillaries and continues recognizing the ACE2 located in the different organs within the blood circulation. As a result, organs expressed with ACE2 may get infected and damaged by the activated immune system, thereby causing the MOF. **B** A hypothesis of how SARS-CoV-2 may trigger ferroptosis. After incubation period, the invading SARS-CoV-2 causes cytotoxic effect to multiple organs. Due to the infection, a plethora of transferrins carrying with Fe^3+^ are recognized by transferrin receptors thereby entering into the cell. Then, divalent metal transporter 1 (DMT1) transformed Fe^3+^ to Fe^2+^, accompanied with iron accumulation in the cell. Hydrogen peroxide (H_2_O_2_), Fe^2+^, and lipid together cause Fenton reaction, producing massive lipid reactive oxygen species (ROS). This can be eliminated by glutathione (GSH) with the help of Glutathione peroxidase 4 (GPX4). However, owing to the iron overload, extensive Fention reaction would generate a large number of lipid ROS, causing cell membrane damage.

It is possible that iron enters and accumulates in the cells, decreasing the serum iron levels. The accumulated iron may trigger the increase in intracellular labile iron (II) pool and Fenton reaction, producing lipid ROS, and lead to ferroptosis^16^. This correlates with the risk of severe and fatal COVID‐19 disease, which is higher in males than in females and also increases with age^49^. Several evidences also reveal the occurrence of significant lymphopenia in a large proportion of COVID-19 patients^2–4,49,62^. Recently, a ferroptosis signature was reported in a case report of COVID-19 patient (48-year-old, male), showing 4-HNE, a reactive breakdown product of the lipid peroxides or oxidized phosphatidylcholine was positive in myocardial tissue staining and in the proximal tubules of acute kidney injury, with decreased lymphocytes in the blood^63^. An in vitro study also showed SARS-CoV-2 suppressed GPX4, the brake of ferroptosis^43^. These studies demonstrated the involvement of ferroptosis in SARS-CoV-2 infection and its potential contribution to the multiple organ damage. Nevertheless, the role of ferroptosis in SARS-CoV-2 infected cells or organs whether it could be a new hope for the treatment of multiple organ involvement of COVID-19 are still unclear (Fig. 1). In spite of the existing findings on the involvement of ferroptosis in SARS-CoV-2 infection, there is no reported study to uncover how does ferroptosis acts in SARS-CoV-2 infection yet. Uncovering the role of ferroptosis in SARS-CoV-2 infection is essential to develop new treatment strategies for COVID-19. New generation of ferroptosis inhibitors such as the improved ferrostatin-1^64,65^ and liproxstatin-1^66^ analogs not only can serve as the tools to evaluate the role of ferroptosis in SARS-CoV-2 infection but also might be potential drug candidates for COVID-19.

## Conclusion

In response to the infection of SARS-CoV-2, iron metabolism dysfunction has been widely documented in a large proportion of COVID-19 patients, and this may cause iron accumulation and overload, triggering ferroptosis in the cells of multiple organs. We hypothesis that ferroptosis is an important cause of multiple organ involvement in COVID-19 and it might serve as a new treatment target. Intracellular cell iron depletion or new generation of ferroptosis inhibitors might be potential drug candidates for COVID-19. We hope this hypothesis may launch a new wave of studies to uncover the association of ferroptosis and SARS-CoV-2 infection in vitro and in vivo.
